# Risk factors for hematological toxicity of chemotherapy for bone and soft tissue sarcoma

**DOI:** 10.3892/ol.2013.1234

**Published:** 2013-03-07

**Authors:** ZHENGXIAO OUYANG, DAN PENG, DIBYA PURUSH DHAKAL

**Affiliations:** Department of Orthopaedics, The Second Xiangya Hospital, Central South University, Changsha, Hunan 410011, P.R. China

**Keywords:** osteosarcoma, chemotherapy, hematological toxicity

## Abstract

The aim of this study was to assess chemotherapy treatment characteristics, neutropenic event occurrence and related risk factors in bone and soft tissue sarcoma patients in China. Knowledge of such risk factors aids healthcare providers in focusing resources on those who are at most risk and targeting prophylactic colony-stimulating factors (CSFs) for those patients. The study included 113 children and adults with different types of sarcoma who had been treated with neoadjuvant chemotherapy for bone and soft tissue sarcoma in order to identify risk factors for hematological toxicity of chemotherapy for bone and soft tissue sarcoma. Risk factors were determined using multivariate logistic regression analysis. Factors such as age <20 years, Karnofsky Performance Status Scale (KPS) score <60, malnutrition, number of previous chemotherapies >3 and combination therapy with >3 drugs were significantly associated with occurrence of grade III/IV neutropenia, suggestive of severe bone marrow suppression. Patients with such characteristics are at most risk of severe bone marrow suppression, and preventing discontinuation of treatment would be valuable for treating patients more effectively.

## Introduction

Current management of osteosarcoma comprises pre- and postoperative chemotherapy and complete surgical removal of all tumor sites (1–3). With this strategy, 5-year overall survival rates of 70% have been reported for patients aged <40 years with non-metastatic, extremity-localized osteosarcoma at diagnosis (4–6). However, anticancer chemotherapies are responsible for numerous adverse events. Among these, hematological toxicity is one of the main reasons for treatment discontinuation. These toxicities decrease production of red blood cells (causing anemia), white blood cells (neutropenia or granulocytopenia) and platelets (thrombocytopenia) which may be life-threatening to the patient. Such complications often result in dose reductions or treatment delays, which may compromise clinical outcome, or even mortality (7–12). Preventing discontinuation of treatment would be valuable for treating patients more effectively. Much research has shown that the hematological toxicity of chemotherapy is based on the regimen and drug dose (13), but 40% of the patients who received high-dose chemotherapy did not experience severe bone suppression as grade III/IV leucopenia (14). It may be considered that the regimen and dosage are not the only risk factors for severe bone marrow suppression. To identify other risk factors for hematological toxicity of chemotherapy for bone and soft tissue sarcoma, 113 patients admitted to the Second Xiangya Hospital of Central South University, China, and treated with consistent neoadjuvant chemotherapy were studied retrospectively. The aim of the study was to decrease the occurrence of hematological toxicity following chemo-therapy and increase the survival rate. The study was approved by the Ethics Committee of the Department of Orthopaedics, The Second Xiangya Hospital, Central South University, Changsha, Hunan, China.

## Materials and methods

### Patients

The present study included 113 children and adults who had been treated with neoadjuvant chemotherapy following the diagnosis of bone and soft tissue sarcoma between June 2007 and April 2012. .Written informed patient consent was obtained from the patients. The mean follow-up period was 29.6 months. The patient characteristics are shown in Table I. In the current study, severe bone marrow suppression was mainly indicated by grade III/IV neutropenia or thrombocytopenia, and the number of patients with grade III/IV anemia was relatively rare. Notably, all patients who experienced grade III/IV neutropenia also experienced grade III/IV thrombocytopenia, but patients with grade III/IV thrombocytopenia did not often experience grade III/IV neutropenia. The 113 patients were therefore divided into two groups (A and B) based on clinical evidence of grade III/IV neutropenia according to World Health Organization (WHO) criteria for hematological toxicity.

### Chemotherapy

**Figure 1:**
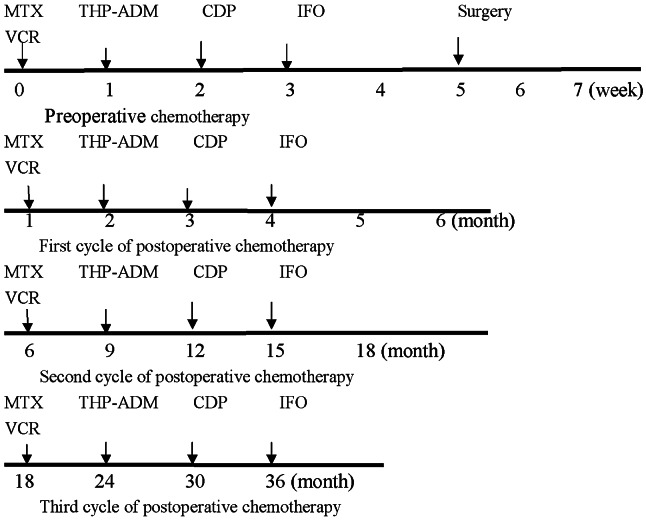
Chemotherapy regimen. MTX, methotrexate; VCR, vincristine; THP-ADM, pirarubicin; CDP, cisplatin; IFO, cyclofosfamide.

All four drugs active against osteosarcoma, cisplatin (CDP), pirarubicin (THP-ADM), methotrexate (MTX) and cyclofosfamide (IFO), were employed. Treatment was performed according to the protocol used at the time of enrollment with adjustment for Chinese racial characteristics. Chemotherapy consisted of 1 cycle of MTX (12 g/m^2^/day; 1 day), THP-ADM (40 mg/m^2^/day; 3 days), CDP 100 mg/m^2^/day; 1 day) and IFO (3 g/m^2^/day; 5 days) preoperatively and 3 cycles postoperatively (Fig. 1). MTX was administered as a 4 h infusion with 11 doses of leucovorin (folinic acid) as rescue (8 mg/m^2^) every sixth hour, beginning 24 h after starting the MTX infusion. Vincristine (VCR; 1.4 mg/m^2^) was delivered two days after the MTX. IFO was in combination with an equal amount of mesna. All drugs were given as single agents.

Complete blood counts and renal and liver function were monitored before each chemotherapy cycle and following infusion. The blood count was monitored twice a week starting on day 1-2 from the start of chemotherapy. No dose reductions were allowed and if the absolute granulocyte count was ≤1,000/*μ*l (500 for MTX cycles) and/or the platelet count was ≤100,000/*μ*l (60,000 for MTX cycles), chemotherapy was delayed until recovery. Granulocyte colony-stimulating factor (G-CSF) and IL-11 support was given according to ASCO guidelines (1994). Component blood transfusion was used as a favorable measure in cases of severe marrow suppression.

### Statistical analysis

The potential significance of age at the diagnosis of cancer (<20), gender (female), malnutrition, Karnofsky Performance Status (KPS) score (<60), leukopenia before chemotherapy (<4.0×10^9^/l), tumor staging (III), lung metastasis, the number of previous chemotherapies (>3) and combination chemotherapy of >3 drugs were evaluated. For univariate analysis, Pearson’s χ^2^ test and one-way ANOVA were used. Factors were kept in the model if the P-value was <0.05. The analysis was performed with SPSS 18.0 software (SPSS Inc. Chicago, IL, USA). For multivariate analysis, factors which were statistically significant were included in the multivariate logistic regression analysis.

## Results

### Patients

In 113 patients, 68 patients (group A) experienced grade III/IV hematological toxicity and the probability of occurrence was 60.18%. Three of the patients among the remainder did not experience any episode of bone marrow suppression. The number of patients with anemia was low. There was no treatment-related mortality (Table II).

### Statistical analysis

**Figure 2:**
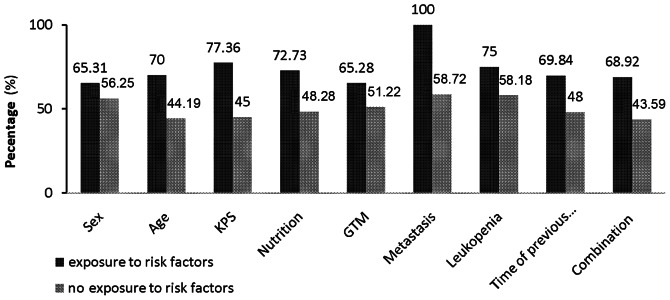
Correlation between potential risk factors and the probability of grade III/IV neutropenia. KPS, Karnofsky Performance Status; GTM, tumor staging.

Univariate analysis revealed correlations similar to those shown using multivariate analysis. Fig. 2 shows the correlation between potential risk factors and the occurrence of grade III/IV neutropenia. For univariate analysis (Table III), the results of the Pearson χ^2^ test are consistent with those of the one-way ANOVA analysis (results not shown). Factors such as age (<20), KPS score (<60), malnutrition, number of previous chemotherapy regimens >3, leucopenia before chemotherapy (<4.0×10^9^/l) and combination chemotherapy with >3 drugs were associated with the occurrence of grade III/IV neutropenia. In the multivariate analysis (Table IV), leucopenia before chemotherapy (<4.0×10^9^/l) was not statistically significant.

## Discussion

In this retrospective study it was found that age <20 years, KPS score <60, malnutrition, >3 previous chemotherapies and combination therapy of >3 drugs were significantly associated with the occurrence of severe bone marrow suppression, mainly indicated by grade III/IV neotropenia. The degree of myelosuppression with relative dose instensity in the same or similar regimens varied greatly, making it difficult to determine the actual risk of neutropenic complications associated with common chemotherapy regimens (15). Treatment dose intensity has also been less consistent, making it difficult to interpret differences in reported toxicity or treatment efficacy. This, in the present study, the chemotherapy regimen was consistent but the dosages of drugs have not been included.

Patients younger than 20 years appear to be more vulnerable to the adverse effects of chemotherapy treatment for bone and soft tissue sarcoma. Ten studies found higher age to be a general risk factor for the development of severe neutropenia (16–22) and other neutropenic complications (23–25). Since older patients are often treated with lower chemotherapy doses to minimize the occurrence of neutropenic complications, advanced age is a particularly important independent risk factor. These studies mainly focused on tumors of non-Hodgkin’s lymphoma (NHL) and breast cancer, however, and the number of patients with bone and soft tissue sarcoma were rare. Bone malignancy, especially osteosarcoma, often occurs in young adolescents. This difference could be due to the inhibition of the hematopoietic system by drugs, especially high-dose Methotrexate (HD-MTX) and THP-ADM. This can be accentuated in younger children whose immature hematopoietic system as well as their great capability for proliferation and differentiation of hematopoietic stem cells makes them more susceptible to the toxicity of chemotherapy. It is possible that higher levels of drugs in tissues and blood occur in younger patients because of their higher percentage of body fat.

In developing countries, due to uneven economic development and imperfect health care systems, malnutrition is common. In malnourished patients, due to poor physical fitness, the toxicity of chemotherapy is tolerated in varying degrees which may be the reason for the higher probability of occurrence of severe bone marrow suppression.

KPS is widely used to quantify the functional status of cancer patients. Studies have shown that, in addition to age, poor performance status is a significant risk factor for chemotherapy-induced neutropenia (26,27). Physiological age or frailty may be a more accurate predictor of risk than chronological age, especially in older patients (28). In the present study, patients with a lower KPS score (<60) were more likely to experience severe bone suppression than those above 60.

The findings suggest that chemotherapy is better tolerated by patients with >3 previous chemotherapies compared with those who received it once or twice. Peripheral neuropathy manifests with paresthesia and hearing loss for high frequencies due to CDP at cumulative doses of 300-600 mg/m^2^(29). It is possible that hematological toxicity results from accumulation of the drug.

The intensity of specific chemotherapy regimens is one of the primary determinants of the risk for severe neutropenia, with some regimens being more myelosuppressive than others (30). High IFO dose or the use of etoposide in treating patients with NHL (16,31) and high anthracycline doses in treating patients with early-stage breast cancer (19,32) have all been identified as significant predictors for severe neutropenia and febrile neutropenia (FN). The combination of four drugs (HD-MTX, THP-ADM, CDP, IFO) in the present study showed an increasing risk of severe bone marrow suppression compared with a three-drug regimen. In light of this, the development of equally effective but less intensive regimens for patients whose disease carries a better prognosis is highly desirable. Ongoing clinical trials are investigating this strategy.

In 1978, Rosen introduced neoadjuvant (preoperative) chemotherapy (33). The goals of neoadjuvant chemotherapy, besides the eradication of micrometastasis, include the destruction of primary tumor cells with reduction of tumor burden and the possibility to evaluate the histologic response to preoperative chemotherapy. At present, neoadjuvant (preoperative) chemotherapy followed by definitive resection with subsequent adjuvant (postoperative) chemotherapy is the well-established approach to the treatment of localized osteosarcomas. Chemotherapy may eradicate the micrometastatic disease that is believed to be present in the majority of patients with clinically resectable cancer. Almost half of young adult survivors of childhood cancer have at least one major adverse outcome as a result of their cancer therapy (34). Chemotherapy-induced bone marrow suppression is the major dose-limiting toxicity of systemic cancer chemotherapy and it is associated with substantial morbidity, mortality and costs. The purpose of the present study was to find the risk factors of severe bone marrow suppression, which may contribute to improve clinical outcomes. The use of such risk factors would be to identify the patients with bone and soft tissue sarcoma who are at greatest risk of neutropenia and target prophylactic colony-stimulating factors (CSFs) to those patients. This should help providers focus resources on those who are at most risk. The present study results, however, are limited by the retrospective design, small population studied and the various risk factors and methods used. Also it is reported that pretreatment WBC counts are predictive of both FN and a relative dose intensity <85% in patients with early-stage breast cancer (15,20). Pretreatment hemoglobin levels <12 g/dl are also predictors of severe neutropenia or FN in cycle 1 (20). In the present study, leukopenia before chemotherapy was of no significance. To overcome these limitations, a prospective registry of different tumor types has been designed, which should make it possible to develop accurate and valid risk models for severe bone suppression in chemotherapy.

## Figures and Tables

**Table I:** Patient characteristics.

Characteristics	No.
Patients	113
Gender	
Male	40
Female	73
Median age (range)	16 (7–39)
Site	
Femur	31
Tibia	25
Humerus	14
Pelvis	15
Knee	12
Elbow	4
Shoulder	9
Other	3
Histology	
Osteosarcoma	63
Malignant fibrous histiocytoma	17
Liposarcoma	12
Synovial sarcoma	10
Rhabdomyosarcoma	5
Leiomyosarcoma	6

**Table II:** Number of patients and the probability of bone marrow suppression.

Bone marrow suppression	No. of patients	Probability (%)
Neutropenia (grade I/II)	45	39.82
Neutropenia (grade III/IV)	68	5.31
Thrombocytopenia (grade I/II)	6	60.18
Thrombocytopenia (grade III/IV)	13	11.50
Anemia (grade I/II)	11	9.73
Anemia (grade III/IV)	7	6.19

**Table III:** Results of univariate analysis (Pearson’s χ^2^ test).

Potential factors	Group A (n)	Group B (n)	Probability (%)	χ^2^	OR	OR 95% CI	P-value
Gender							
Male	36	28	56.25				
Female	32	17	65.31	0.950	1.464	0.679–3.156	>0.05
Age							
<20	49	21	70.00	7.407	0.339	0.154-0.747	<0.01
≥20	19	25	44.19				
KPS score							
<60	41	12	77.36	12.296	0.239	0.105–0.544	<0.05
≥60	27	33	45.00				
Tumor staging (Enneking)							
G1/2T1/2M1	47	25	65.28				
G1/2T1/2M0	21	20	51.22	2.154	1.790	0.820–3.911	>0.05
Nutrition							
Good	40	15	72.73	7.043	2.857	1.302–6.269	<0.01
Poor	28	30	48.28				
Lung metastasis							
Metastatic	4	0	100.00	2.744	0.587	0.502–0.687	>0.05
Local	64	45	58.72				
Leucopenia before chemotherapy							
Abnormal	36	12	75.00	7.651	3.094	1.370–6.985	<0.01
Normal	32	33	49.23				
No. of previous chemotherapies							
>3	44	19	69.84	5.549	2.509	1.158–5.543	<0.05
0–3	24	26	48.00				
Combination chemotherapy							
1–3	17	22	43.59				
>3	51	23	68.92	6.837	2.870	1.287–6.398	<0.05

KPS, Karnofsky Performance Status.

**Table IV:** Results of multivariate analysis (multivariate logistic regression analysis).

Factors	B	SE	OR	OR 95% CI	Wald	P-value
Age (<20)	−2.151	0.628	0.116	0.034–0.398	11.730	0.001
KPS score (<60)	−2.249	0.611	0.105	0.032–0.349	13.540	0.000
Malnutrition	1.332	0.593	3.787	1.184–12.109	5.040	0.025
Leucopenia before chemotherapy	0.018	0.565	1.019	0.337–3.081	0.001	0.974
No. of previous chemotherapies (>3)	1.245	0.673	3.474	1.004–12.019	3.868	0.049
Combination chemotherapy (>3)	1.815	0.597	6.142	1.905–19.803	9.234	0.004

KPS, Karnofsky Performance Status.
